# Dose-dependent pro- or anti-fibrotic responses of endometriotic stromal cells to interleukin-1β and *tumor necrosis factor* α

**DOI:** 10.1038/s41598-020-66298-x

**Published:** 2020-06-11

**Authors:** Sachiko Matsuzaki, Jean-Luc Pouly, Michel Canis

**Affiliations:** 1 CHU Clermont-Ferrand, Chirurgie Gynécologique, Clermont-Ferrand, France; 2 Université Clermont Auvergne, Institut Pascal, UMR6602, CNRS/UCA/SIGMA, Clermont-Ferrand, France; 3 CHU Clermont-Ferrand, Chirurgie Gynécologique, 1, Place Lucie et Raymond Aubrac, 63003 Clermont-Ferrand, France

**Keywords:** Molecular biology, Diseases, Molecular medicine, Pathogenesis

## Abstract

Endometriosis are characterized by dense fibrous tissue. Numerous studies have investigated roles of inflammation on the pathophysiology of endometriosis. However, the interplay of inflammation and fibrosis remains to be clarified. Here we show that low levels of interleukin-1β (IL-1β) and tumor necrosis factor-alpha (TNFα) promoted a fibrotic phenotype, whereas high levels of IL-1β and TNFα inactivated the fibrotic phenotype of endometriotic stromal cells (Ectopic-ES). IL-1β 10 pg/mL and TNFα 100 and 1,000 pg/mL had minimal effects, whereas the highest dose of IL-1β (100 pg/mL) significantly decreased collagen gel contraction in Ectopic-ES. Furthermore, in Ectopic-ES, low levels of IL-1β (1 pg/mL) and/or TNFα 10 pg/mL significantly increased Col I mRNA expression, whereas higher doses of IL-1β (10 and/or 100 pg/mL) and/or TNFα (100 and/or 1,000 pg/mL) significantly decreased Col I and/or αSMA mRNA expression and the percentage of cells with Col I + and/or αSMA + stress fibers. In contrast, in either menstrual endometrial stromal cells of patients with endometriosis or those of healthy women, varying doses of IL-1β and/or TNFα had no significant effects on either Col I or αSMA mRNA/protein expression. The present findings bring into question whether we should still continue to attempt anti-inflammatory treatment strategies for endometriosis.

## Introduction

Endometriosis, particularly deep infiltrating endometriosis and ovarian endometriosis, is histologically characterized by the presence of dense fibrous tissue^1–3^. Previous studies including those from our group demonstrated that knowledge of the cellular and molecular mechanisms of fibrosis is indispensable for the development of strategies to prevent and treat this condition^4–12^.

Acute inflammatory reactions play an important part in triggering fibrosis in many different organ systems^13^. Low-grade but persistent inflammation is also thought to contribute to the progression of fibrosis^13^. In many fibrotic disorders, a persistent inflammatory trigger is crucial to the activation of the wound-healing process that leads to fibrosis^13^. Endometriosis has been considered to be an immune-mediated chronic inflammatory disorder^14,15^. Numerous studies have investigated the role of immune-mediated chronic inflammation on the pathophysiology of endometriosis^14,15^ and attempted to evaluate various anti-inflammatory drugs, including tumor necrosis factor-alpha TNFα inhibitors^16–18^ and cyclooxygenase-2 (Cox-2) inhibitors^19–21^, in endometriosis. However, the interplay between inflammation and fibrosis at the cellular and molecular levels in endometriosis pathophysiology remains to be clarified. Studies have suggested that repeated tissue injury and repair caused by recurrent menstrual bleeding induce inflammation, resulting in fibrosis in endometriosis^8,9^. However, endometrial repair is normally scarless^22^. It is not clear why repeated tissue injury and repair caused by recurrent menstrual bleeding do not induce fibrosis in the endometrium, whereas they do in endometriosis. According to the implantation theory, which is one of the most supported theories of the pathogenesis of endometriosis, endometriosis originates from retrograde menstruation of endometrial tissue, which may then implant into the peritoneal cavity^3^. Retrograde menstruation and the presence of endometrial cells within the peritoneal cavity can induce inflammation^23^. In addition, studies have shown that the eutopic endometrium of patients with endometriosis is different in many ways from that of healthy controls^24^. Inflammation may not induce fibrosis in cycling endometrium, but may induce a pro-fibrotic phenotype of endometrial stromal cells of patients with endometriosis after implantation into the peritoneal cavity.

In the present study, we attempted to investigate whether inflammation could trigger fibrosis in endometriosis as well as in menstrual endometrium *in vitro*. IL-1β and TNFα are central inflammation mediators^25^. Numerous studies have demonstrated the involvement of these two proinflammatory cytokines in the pathophysiology of endometriosis^14,15^. Levels of cytokines, including IL-1β and TNFα, *vary* greatly among individuals and depend on pathology. Thus, we investigated the effects of varying doses of IL-1β and/or TNFα (supplementary Note, supplementary Methods, supplementary Results, supplementary Fig. S1) on cell proliferation, cell migration, collagen gel contraction, mRNA and/or protein expression of collagen type I (Col I), matrix metalloproteinase-1 (MMP-1), and alpha smooth muscle actin (αSMA), which are commonly used methods for evaluating fibrosis^4–12^, in endometriotic and menstrual endometrial stromal cells of patients with endometriosis. We included menstrual endometrial stromal cells of healthy fertile women for comparison.

## Results

The results are summarized in Tables 1, 2. There were no significant differences in the effects of varying doses of IL-1β and TNFα on cell migration, collagen gel contraction, Col I, αSMA and MMP-I mRNA and/or protein expression of Ectopic-ES derived from deep infiltrating endometriosis versus ovarian endometriosis.

**Table 1:** Summary of effects of IL-1β and TNFα on cell proliferation, migration and collagen gel contraction in M-ES-healthy, M-ES-endo, and Ectopic-ES.

	M-ES-healthy		M-ES-endo		Ectopic-ES	
	IL-1β (pg/mL)	TNFα (pg/mL)	IL-1β (pg/mL)	TNFα (pg/mL)	IL-1β (pg/mL)	TNFα (pg/mL)
Proliferation	↑ :1–10↓: 50–100	↑	↑ :1–10↓: 50–100	↑	↑	↑
Migration	N.S.	N.S.	N.S.	N.S.	↑	↑
Collagen gel contraction	↓ :10	↓ :100–1000	↑ :10	↑	↓: 100	N.S.

M-ES-healthy: menstrual endometrial stromal cells of healthy fertile women.

M-ES-endo: menstrual endometrial stromal cells of patients with endometriosis.

Ectopic-ES: endometriotic stromal cells.

N.S.: not significant.

According to the results of the present cell proliferation assays, effects of the highest dose of IL-1β (100 pg/mL) on cell migration, collagen gel contraction in M-ES-healthy and M-ES-endo were excluded for further analyses, due to markedly reduced cell viability.

**Table 2:** Summary of effects of IL-1β and TNFα with or without TGF-β1 on expression of Col I, MMP-1 and αSMA in M-ES-healthy, M-ES-endo, and Ectopic-ES.

			M-ES-healthy		M-ES-endo		Ectopic-ES	
			IL-1β (pg /mL) :1–10	TNFα (pg/mL) :10–1000	IL-1β (pg/mL) :1–10	TNFα (pg/mL) :10–1000	IL-1β (pg/mL) :1–100	TNFα (pg/mL) :10–1000
Col 1	mRNA	TGF-β1(−)	N.S.	N.S.	N.S.	N.S.	↑: 1 ↓: 100	↑: 10 ↓: 1000
		TGF-β1(+)	N.S.	N.S.	↑	↑	↓: 10–100	↓: 100–1000
	protein	TGF-β1(−)	N.S.	N.S.	N.S.	N.S.	↓: 100	↓: 1000
		TGF-β1(+)	N.S.	N.S.	N.S.	N.S.	↓: 100	↓: 1000
MMP1	mRNA	TGF-β1(−)	N.S.	N.S.	N.S.	N.S.	↑: 10–100	↑: 100–1000
		TGF-β1(+)	N.S.	N.S.	N.S.	N.S.	N.S.	N.S.
αSMA	mRNA	TGF-β1(−)	N.S.	N.S.	N.S.	N.S.	↓: 10–100	↓: 100–1000
		TGF-β1(+)	N.S.	N.S.	N.S.	N.S.	↓: 10–100	↓: 100–1000
	protein	TGF-β1(−)	N.S.	N.S.	N.S.	N.S.	↓: 10–100	↓: 100–1000
		TGF-β1(+)	N.S.	N.S.	N.S.	N.S.	↓: 10–100	↓: 100–1000

Col I: collagen type I.

MMP-1: matrix metalloproteinase-1.

αSMA: alpha smooth muscle actin.

M-ES-healthy: menstrual endometrial stromal cells of healthy fertile women.

M-ES-endo: menstrual endometrial stromal cells of patients with endometriosis.

Ectopic-ES: endometriotic stromal cells.

N.S.: not significant.

According to the results of the present cell proliferation assays, effects of the highest dose of IL-1β (100 pg/mL) on cell migration, collagen gel contraction in M-ES-healthy and M-ES-endo were excluded for further analyses, due to markedly reduced cell viability.

### Effects of IL-1β and TNFα on cell proliferation of endometrial and endometriotic stromal cells

**Figure 1:**
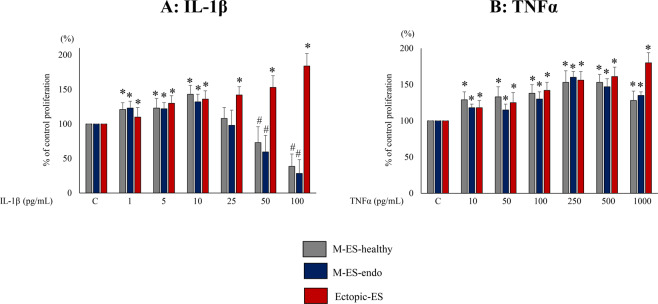
Effects of IL-1β (**A**) or TNFα (**B**) on cell proliferation of M-ES-healthy, M-ES-endo, and Ectopic-ES. Cells were incubated for 48 h at the indicated concentrations. Percent cell proliferation was calculated as percent of vehicle control after 48-h treatment. *Higher cell proliferation (p < 0.05) versus control (vehicle alone). #Lower cell proliferation (p < 0.05) versus control (vehicle alone). Numerical values are presented as the mean ± SD. C: control (vehicle alone). M-ES-healthy: n = 8. M-ES-endo: n = 8. Ectopic-ES: n = 16.

IL-1β doses of 1–10 pg/mL significantly increased cell proliferation of M-ES-healthy and M-ES-endo, whereas IL-1β 50–100 pg/mL significantly decreased cell proliferation of M-ES-healthy and M-ES-endo, compared with vehicle-treated control (Fig. 1A). TNFα 10–1,000 pg/mL significantly increased cell proliferation of M-ES-healthy and M-ES-endo (Fig. 1B). In contrast, both IL-1β (Fig. 1A) and TNFα (Fig. 1B) significantly increased cell proliferation of Ectopic-ES dose-dependently. The highest dose of IL-1β (100 pg/mL) induced significantly more proliferation of Ectopic-ES derived from ovarian endometriosis compared to that of deep infiltrating endometriosis (Supplementary Fig. S2).

### Effects of IL-1β and TNFα on cell migration of endometrial and endometriotic cells

**Figure 2:**
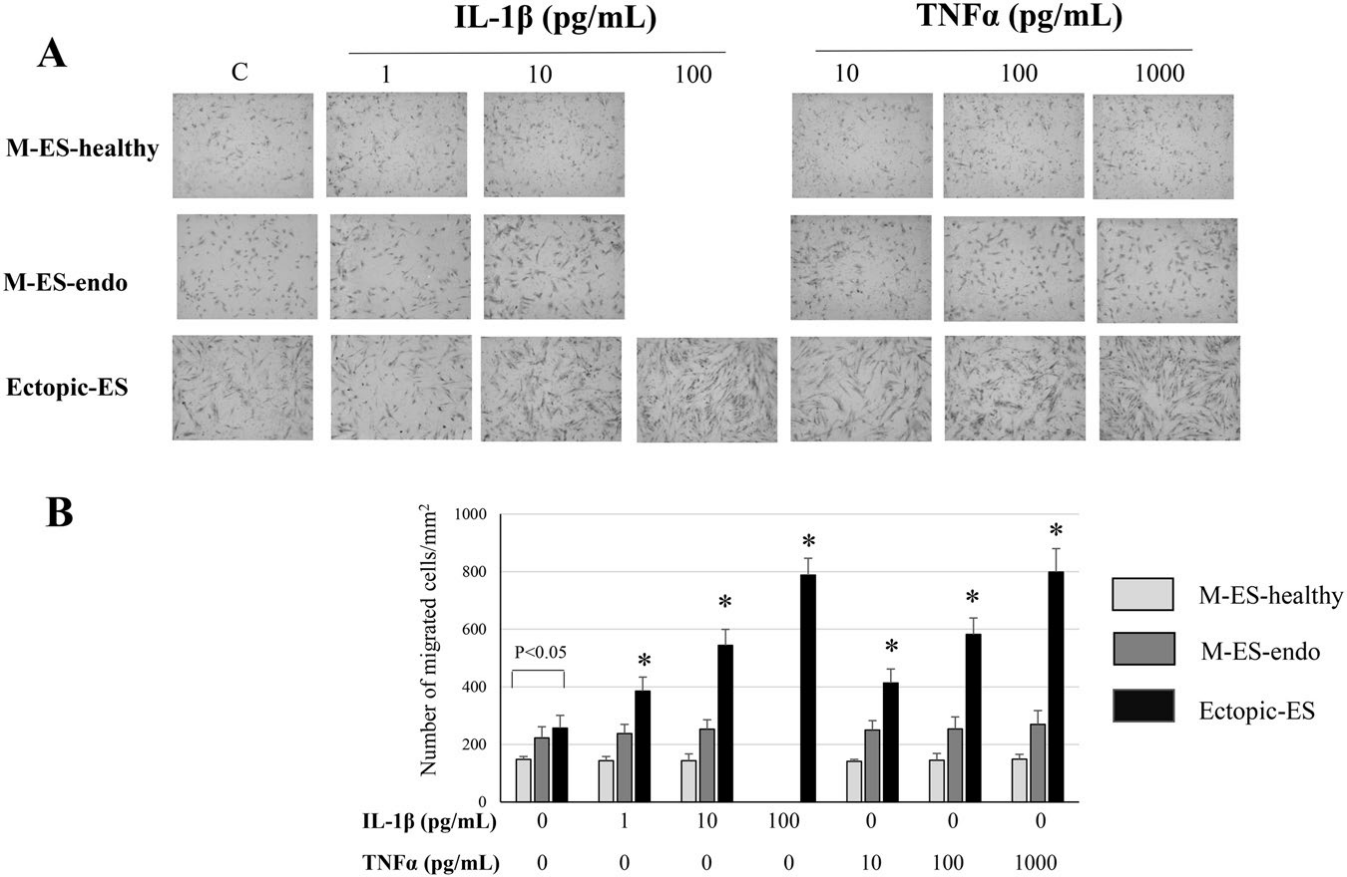
Effects of IL-1β and TNFα on cell migration of M-ES-healthy, M-ES-endo, and Ectopic-ES. (**A**) Representative photomicrographs of cell migration in M-ES-healthy, M-ES-endo, and Ectopic-ES treated with either IL-1β or TNFα at indicated concentrations, or vehicle alone. (**B**) Number of migrated cells/mm^2^ in M-ES-healthy, M-ES-endo, and Ectopic-ES treated with IL-1β or TNFα at indicated concentrations, or vehicle alone. Results are presented as the mean + SD. *p < 0.05: versus control (vehicle alone) within the same group. C: control (vehicle alone). Effects of the highest dose of IL-1β (100 pg/mL) on cell migration in M-ES-healthy and M-ES-endo were excluded for further analyses, due to markedly reduced cell viability. M-ES-healthy: n = 6. M-ES-endo: n = 10. Ectopic-ES: n = 18.

At basal levels, cell migration of Ectopic-ES was significantly higher than that of M-ES-healthy (Fig. 2A,B). We observed no significant effects of either IL-1β or TNFα on cell migration of M-ES-healthy or M-ES-endo (Fig. 2A,B). In contrast, varying doses of IL-1β (1, 10, and 100 pg/mL) and TNFα (10, 100, and 1,000 pg/mL) significantly increased cell migration of endometriotic stromal cells dose-dependently (Fig. 2A,B).

### Effects of IL-1β and TNFα on collagen gel contraction of endometrial and endometriotic cells

**Figure 3:**
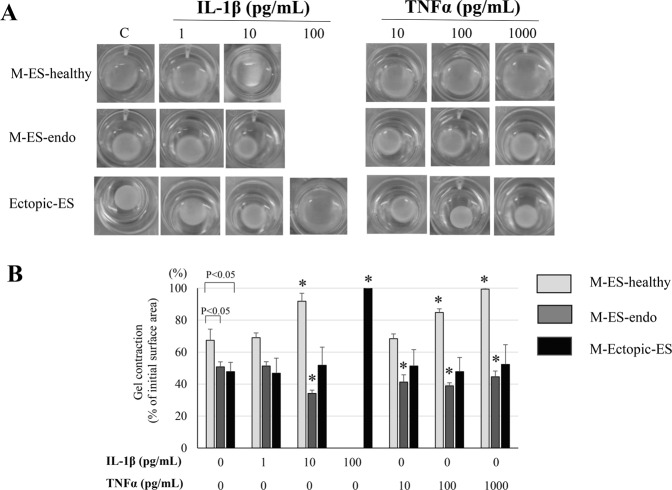
Effects of IL-1β and TNFα on collagen gel contraction of M-ES-healthy, M-ES-endo, and Ectopic-ES. (**A**) Representative photomicrographs of contracted gels taken at 24 h in M-ES-healthy, M-ES-endo, and Ectopic-ES treated with IL-1β or TNFα at the indicated concentrations, or vehicle. (**B**) Collagen gel contraction at 24 h in M-ES-healthy, M-ES-endo, and Ectopic-ES treated with IL-1β or TNFα at the indicated concentrations, or vehicle alone. *p < 0.05: versus control (vehicle alone) within the same group. C: control (vehicle alone). Numerical values are presented as the mean + SD. M-ES-healthy: n = 6. M-ES-endo: n = 8. Ectopic-ES: n = 16.

At basal levels, collagen gel contraction of M-ES-healthy was significantly lower than that of M-ES-endo and Ectopic-ES (Fig. 3A,B). In M-ES healthy, IL-1β 10 pg/mL and TNFα 100 and 1,000 pg/mL significantly decreased collagen gel contraction, whereas either IL-1β 1 pg/mL or TNFα 10 pg/mL had no significant effects on collagen gel contraction (Fig. 3A,B). In contrast, in M-ES-endo, IL-1β 10 pg/mL and TNFα 10, 100 and 1,000 pg/mL significantly increased collagen gel contraction, whereas IL-1β 1 pg/mL had no significant effects on collagen gel contraction (Fig. 3A,B). In Ectopic-ES, IL-1β 1 and 10 pg/mL and TNFα 10, 100, and 1,000 pg/mL had no significant effects, whereas IL-1β 100 pg/mL significantly decreased collagen gel contraction (Fig. 3A,B).

### Effects of IL-1β and TNFα with or without TGF-β1 stimulation on mRNA expression of Col I, MMP-1, and αSMA in endometrial and endometriotic stromal cells

#### Col I

**Figure 4:**
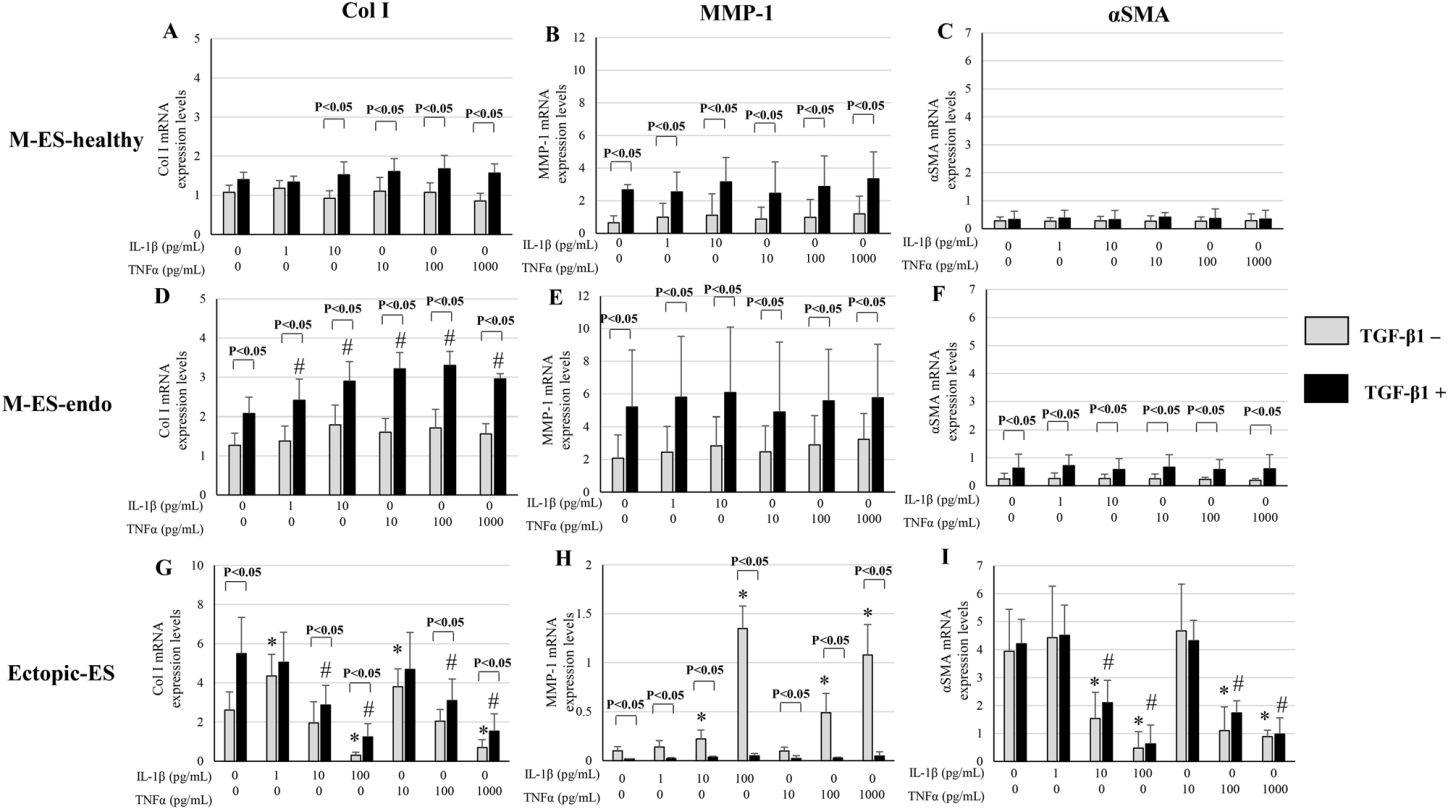
Effects of IL-1β and TNFα with or without transforming growth factor-beta 1 (TGF-β1) on mRNA expression of collagen type I (Col I) (**A**,**D**,**G**), matrix metalloproteinase-1 (MMP-1) (**B**,**E**,**H**), and alpha smooth muscle actin (αSMA) (**C**,**F**,**I**) in M-ES-healthy (**A–C**), M-ES-endo (**D–F**), and Ectopic-ES (**G–I**). Cells were incubated at the indicated concentrations. Expression levels of Col-I, MMP-1, and αSMA mRNAs are given relative to the expression level of the reference gene, glyceraldehyde 3-phosphate dehydrogenase (GAPDH). Numerical values are presented as the mean + SD. *p < 0.05 versus control (vehicle alone). #p < 0.05 versus TGF-β1 alone. Because there were no significant differences in either Col I, MMP-1, or αSMA mRNA expression between vehicle-treated control after 48 h and 96 h, results of vehicle-treated control after 96 h were not shown. Effects of the highest dose of IL-1β (100 pg/mL) on mRNA expression of Col I, MMP-1, and αSMA in M-ES-healthy and M-ES-endo were excluded for further analyses, due to markedly reduced cell viability. M-ES-healthy: n = 8. M-ES-endo: n = 16. Ectopic-ES: n = 22.

In M-ES-healthy, varying doses of IL-1β and/or TNFα with or without TGF-β1 5 ng/mL stimulation had no significant effects on Col I mRNA expression (Fig. 4A, Supplementary Fig. S3A). In M-ES-endo, without TGF-β1 5 ng/mL stimulation, varying doses of IL-1β and/or TNFα had no significant effects on Col I mRNA expression (Fig. 4D, Supplementary Fig. S3D). With TGF-β1 stimulation, varying doses of IL-1β and/or TNFα with TGF-β1 stimulation significantly increased Col I mRNA expression (Fig. 4D, Supplementary Fig. S3D).

In Ectopic-ES, IL-1β 1 pg/mL and/or TNFα 10 pg/mL without TGF-β1 significantly increased, whereas higher doses of IL-1β (100 pg/mL), TNFα (1,000 pg/mL), or combinations (IL-1β 10 pg/mL + TNFα 1,000 pg/mL or IL-1β 100 pg/mL + TNFα 1,000 pg/mL) significantly decreased Col I mRNA expression (Fig. 4G, Supplementary Fig. S3G). When TGF-β1 5 ng/mL was added, IL-1β (10 and100 pg/mL), TNFα (100 and 1,000 pg/mL), or combinations (IL-1β 10 pg/mL + TNFα 1000 pg/mL or IL-1β 100 pg/mL + TNFα 1000 pg/mL) significantly decreased Col I mRNA expression (Fig. 4G, Supplementary Fig. S3G).

#### MMP-1

At basal levels, MMP-1 mRNA expression was significantly different between M-ES-healthy, M-ES-endo, and Ectopic-ES. M-ES-endo showed the highest expression and Ectopic-ES showed the lowest one (p < 0.05).

Varying doses of IL-1β and/or TNFα with or without TGF-β1 5 ng/mL stimulation had no significant effects on MMP-1 mRNA expression in either M-ES-healthy or M-ES-endo (Fig. 4B, E, Supplementary Fig. S3B,E).

In Ectopic-ES, IL-1β 1 pg/mL and/or TNFα 10 pg/mL without TGF-β1 did not significantly influence MMP-1 mRNA expression, whereas higher doses of IL-1β (10 and 100 pg/mL), TNFα (100 and 1,000 pg/mL), or combinations (IL-1β 1 pg/mL + TNFα 100 pg/mL, IL-1β 10 pg/mL + TNFα 1,000 pg/mL, or IL-1β 100 pg/mL + TNFα 1,000 pg/mL) significantly increased MMP-1 mRNA expression (Fig. 4H, Supplementary Fig. S3H).

#### αSMA

At basal levels, αSMA mRNA expression was significantly higher in Ectopic-ES than in M-ES-healthy and M-ES-endo (p < 0.05).

Varying doses of IL-1β and/or TNFα with or without TGF-β1 5 ng/mL stimulation had no significant effects on αSMA mRNA expression in either M-ES-healthy or M-ES-endo (Fig. 4C,F, Supplementary Fig. S3C,F).

In contrast, in Ectopic-ES, higher doses of IL-1β (10 and 100 pg/mL), TNFα (100 and 1,000 pg/mL), or combinations (IL-1β 1 pg/mL + TNFα 100 pg/mL, IL-1β 10 pg/mL + TNFα 1,000 pg/mL, or IL-1β 100 pg/mL + TNFα 1,000 pg/mL) with or without TGF-β1 significantly decreased αSMA mRNA expression compared with vehicle-treated control (Fig. 4I, Supplementary Fig. S3I).

### Effects of IL-1β and TNFα with or without TGF-β1 on Col I protein and αSMA + stress fibers expression in endometrial and endometriotic stromal cells

At basal levels, the percentage of Col I + cells and that of cells with αSMA + stress fibers were significantly higher in Ectopic-ES than those of M-ES-healthy and M-ES-endo (p < 0.05).

**Figure 5:**
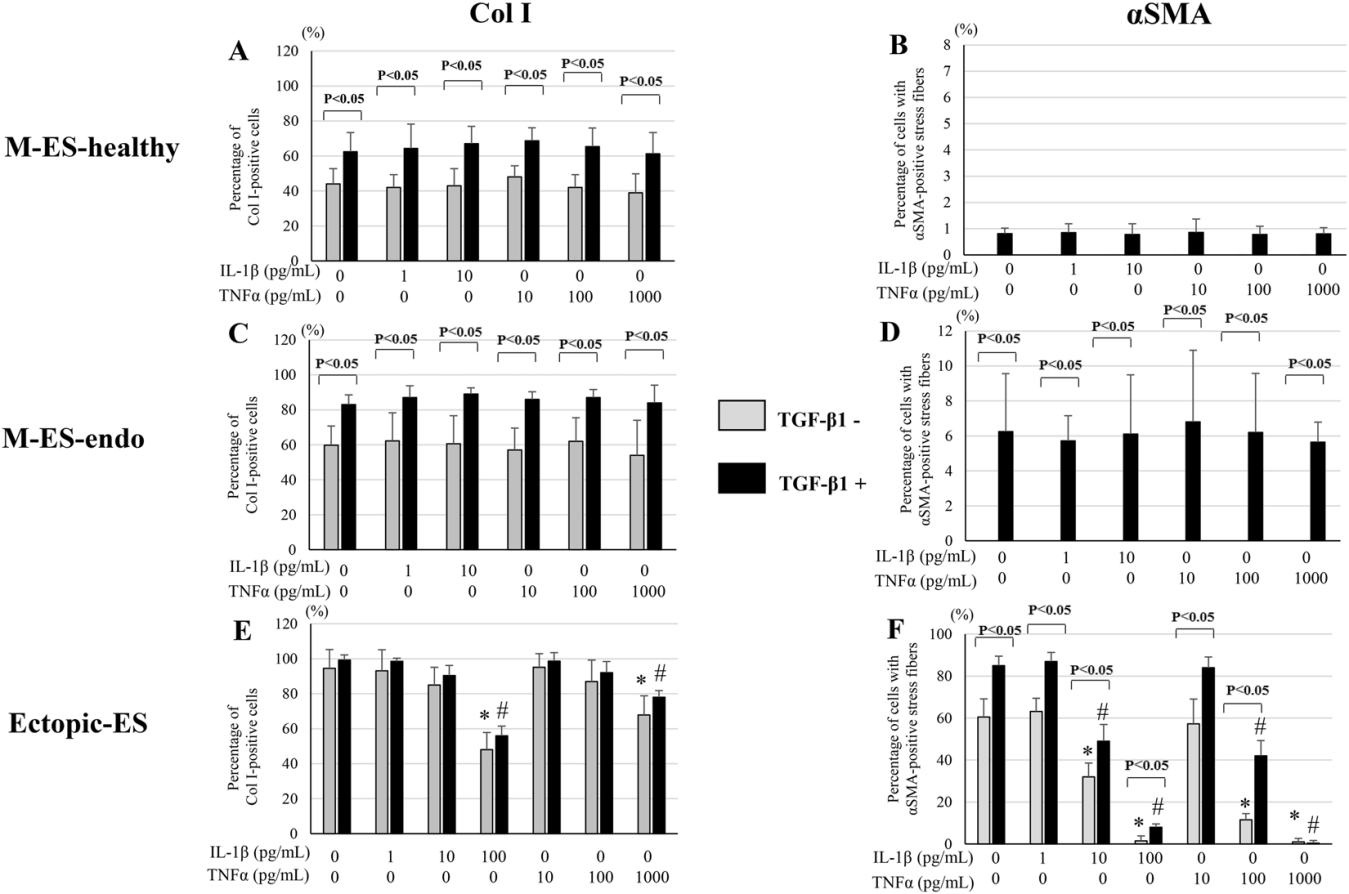
Effects of IL-1β and TNFα with or without transforming growth factor-beta 1 (TGF-β1) on Col I protein expression (**A**,**C**,**E**) and αSMA + stress fibers (**B**,**D**,**F**) in M-ES-healthy (A,B), M-ES-endo (C,D), and Ectopic-ES (E,F). (**A–F**) The percentage of cells with Col I + or αSMA + stress fibers after stimulation with IL-1β or TNFα alone or following TGF-β1 5 ng/mL stimulation in M-ES-healthy, M-ES-endo, and Ectopic-ES. *p < 0.05: versus control (vehicle alone). #p < 0.05 versus TGF-β1 alone. Cells were incubated at the indicated concentrations. Numerical values are presented as the mean ± SD. Because there were no significant differences in either Col I or αSMA protein expression between vehicle-treated control after 48 h and 96 h, results of vehicle-treated control after 96 h were not shown. Effects of the highest dose of IL-1β (100 pg/mL) on protein expression of Col I and αSMA in M-ES-healthy and M-ES-endo were excluded for further analyses, due to markedly reduced cell viability. M-ES-healthy: n = 8. M-ES-endo: n = 16. Ectopic-ES: n = 22.

**Figure 6:**
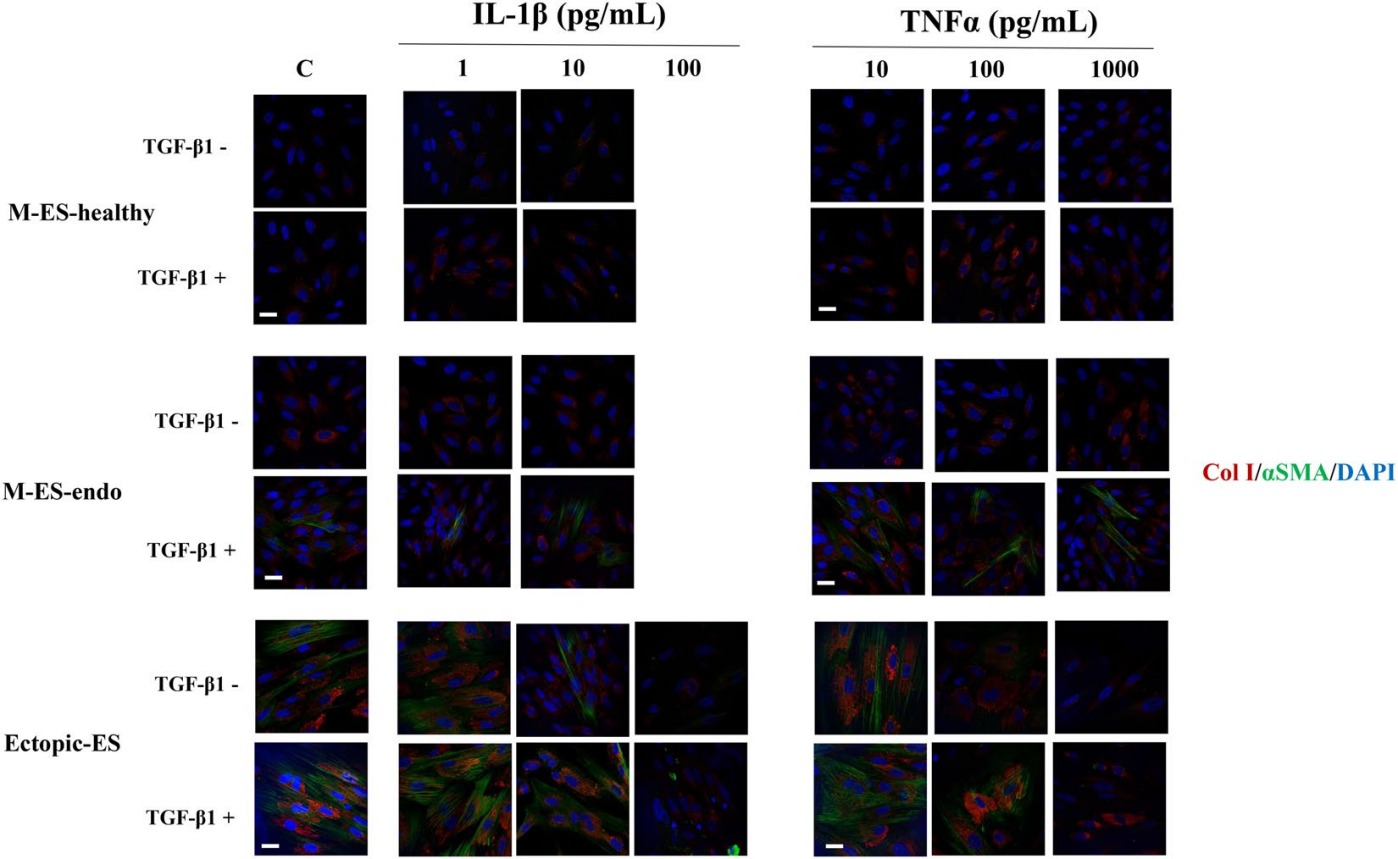
Representative photomicrographs of double immunofluorescence staining for Col I and αSMA in M-ES-healthy, M-ES-endo, and Ectopic-ES after stimulation with IL-1β or TNFα alone or following TGF-β1 5 ng/mL stimulation. Scale bar: 50 μm. Effects of the highest dose of IL-1β (100 pg/mL) on protein expression of Col I and αSMA in M-ES-healthy and M-ES-endo were excluded for further analyses, due to markedly reduced cell viability.

In either M-ES-healthy or M-ES-endo, varying doses of IL-1β and/or TNFα with or without TGF-β1 stimulation had no significant effects on either the percentage of Col I + cells (Fig. 5A,C, Fig. 6, Supplementary Fig. S4A, S4C, Fig. S5) or that of cells with αSMA + stress fibers (Fig. 5B,D, Fig. 6, Supplementary Fig. S4B,D, Fig. S5).

In contrast, in Ectopic-ES, higher doses of IL-1β (10 and/or100 pg/mL), TNFα (1,000 pg/mL), or combinations (IL-1β 1 pg/mL + TNFα 1,000, IL-1β 10 pg/mL + TNFα 1,000 pg/mL and/or IL-1β 100 pg/mL + TNFα 1,000 pg/mL) with or without TGF-β1 5 ng/mL stimulation significantly decreased the percentage of Col I + cells and/or αSMA + stress fibers (Fig. 5E,F, Fig. 6, Supplementary Fig. S4E,F, S5).

## Discussion

The present study provided further insights on the distinct characteristics of the menstrual endometrium of patients with endometriosis compared with those of healthy fertile women and further supports the concept that endometrium of patients with endometriosis may differ biochemically from that of patients without endometriosis^24^. MMP-1 breaks down interstitial Col I, II, and III and is only expressed during the perimenstrual period in endometrial stromal cells^26,27^. A baboon model of endometriosis showed that the presence of ectopic lesions enhanced expression of MMP-1 mRNA of the eutopic endometrium^28^. In the present study, we showed that basal MMP-1 mRNA levels were significantly higher in M-ES-endo than in M-ES-healthy. Higher MMP-1 of M-ES-endo could facilitate implant into the peritoneum. The present results suggest that inflammation during menstruation could simulate collagen gel contraction of M-ES-endo. Previous studies have shown that stiffness precedes fibrosis^29,30^, and that extracellular matrix (ECM) stiffening induces latent TGF-β1 activation^31^. After implanting into the peritoneum in a Col I-rich microenvironment, cyclical menstrual inflammation may gradually promote ECM stiffening, which may subsequently activate latent TGF-β1. In our previous study^32^, we showed that a long culture duration is required to promote endometrial stromal cells to differentiate into myofibroblasts and for Col I synthesis. ECM stiffening along with TGF-β1 activation may gradually increase Col I synthesis, myofibroblast differentiation, and cell proliferation of M-ES-endo after implantation over time. In contrast, lower MMP-1 in M-ES-healthy may have less potential to implant into the peritoneum. Furthermore, the present results suggest that cyclical menstrual inflammation may decrease rather than increase collagen gel contraction of M-ES-healthy, resulting in prevention of ECM stiffening over time. Further studies that investigate underlying molecular mechanisms of the distinct response of M-ES-endo to cyclical menstrual inflammation in collagen gel contraction should provide further insights on pathophysiology of endometriosis

The present study suggested that low levels of inflammation may gradually promote myofibroblast differentiation and collagen deposition, so ECM stiffening may continue over time, resulting in progression of fibrosis. In contrast, varying doses of IL-1β and/or TNFα had minimal effects on αSMA and Col I expression in both M-ES-endo and M-ES-healthy. Furthermore, basal levels of MMP-1 mRNA as well as those after treatment with IL-1β and/or TNFα were significantly lower in Ectopic-ES compared with M-ES-endo and M-ES-healthy. In addition, TGF-β1 stimulation increased MMP-1 mRNA expression in both M-ES-endo and M-ES-healthy, whereas it decreased MMP-1 mRNA expression in Ectopic-ES. The present study did not measure MMP-1 activity. However, the present immunofluorescence staining results showed a much higher percentage of Col I positivity in Ectopic-ES compared with M-ES-endo and M-ES-healthy, suggesting much higher collagen deposition in Ectopic-ES. These results support our previous findings that in the endometrium, collagen synthesis and degradation may be precisely balanced to maintain proper tissue architecture^7^. However, in endometriosis, impaired collagen degradation may result in increased collagen deposition^7^. Our present findings and previous findings^7^ suggest that myofibroblast differentiation and increased collagen deposition through low-grade inflammation may be an important molecular mechanism of fibrosis in endometriosis.

The present results also suggest that when excessive inflammation dominates, collagen degradation, decreased myofibroblast differentiation, and increased cell migration may continue, resulting in proteolytic ECM remodeling and tissue destruction of endometriosis. These findings suggest distinct biphasic responses to inflammation in endometriotic tissues, whereas inflammation may minimally affect the endometrium, resulting in a stable microanatomy of the ECM of the endometrium after cyclical tissue injury and repair. Previous studies showed conflicting data regarding the effects of IL-1β and/or TNFα with or without TGF-β1 on collagen expression/synthesis of fibroblasts and on the differentiation of fibroblasts to myofibroblasts^33–41^. The present study may partly explain these conflicting data, as (1) fibroblasts from different origins show different phenotypical responses toward inflammation^41,42^, and (2) fibroblasts show different phenotypical responses to varying levels of inflammation. The present findings may also partly explain why repeated tissue injury and repair caused by recurrent menstrual bleeding do not induce fibrosis in the endometrium, whereas they could do so in endometriosis. Tissue injury causes the immediate onset of acute inflammation followed by resolution of the inflammatory response, which is essential for successful tissue repair^43^. In the endometrium, tissue regeneration is self-limiting, reaching an equilibrium of a stable ECM microanatomy and vascularization^44^. However, defects may persist in tissue repair of endometriosis, resulting in fibrosis. When the defect persists in tissue repair, continuation of fibrosis and/or inflammation can result in distinct ECM remodeling outcomes^45^. When the myofibroblast response dominates, collagen deposition and stiffening may continue^45^. In contrast, when (sub)acute inflammation dominates, continuous proteolytic ECM remodeling and collagen degradation may ultimately destroy tissue^45^.

When patients with endometriosis require medical treatment, already developed endometriosis characterized by dense fibrotic tissues is targeted in general. In such fibrotic tissues, the myofibroblast response may dominate. The present *in vitro* findings suggested that anti-inflammatory treatment in endometriosis may reduce inflammation and subsequently increase fibrosis. Previous animal and clinical studies might support the present *in vitro* findings. Anti-TNFα treatment in a baboon model of endometriosis showed a decreased active red lesion surface area and an increased number and surface area of fibrotic white and black lesions^16^ suggesting that anti-TNFα treatment might induce a myofibroblast response. A randomized clinical trial showed no effects of anti-TNFα treatment on deep endometriosis-associated pain^18^. Various anti-TNFα agents have been used in clinical practice for the management of inflammatory bowel disease (IBD) for the last 20 years^46,47^. However, therapeutic strategies to block TNFα to prevent fibrostenosis in Crohn’s disease have only been successful in animal models^48^. Clinical studies have suggested that anti-TNFα treatment in patients with Crohn’s disease does not prevent fibrostenosis but rather promotes ECM deposition, result in resolution of fistulae^49,50^. Patients with inflammatory-stage fibrotic disease are most likely to respond, while patients with noninflammatory fibrosis might experience deleterious effects^42^. Administration of COX-2 inhibitors in the early phase of inflammation yields an anti-inflammatory effect. However, inhibition of COX-2 by nonsteroidal anti-inflammatory drugs (NSAIDs), if used for more than 48 h, causes inhibition of anti-inflammatory mediators^51,52^, and thus prolongs chronic inflammation and activates fibrosis of the kidneys^53^, lungs^54^, intestines^55^, and muscles^56^, as COX-2 is an important anti-fibrotic enzyme^54^. In our previous animal experiments, we showed that use of a selective COX-2 inhibitor can prevent initial development of ectopic implants in our rat model of endometriosis^20^. However, when selective COX-2 inhibitor treatment was started after the establishment of ectopic implants, all ectopic implants remained detectable after 4 weeks of treatment^20^.

Therapeutic approaches to inflammation have focused on suppressing, blocking, or inhibiting proinflammatory mediators of inflammation^57^. The present findings bring into question whether we should still continue to attempt anti-inflammatory treatment strategies in endometriosis, because both the present findings and previous findings suggest that such traditional therapeutic strategies may promote fibrosis, resulting in progression of endometriosis^58^. Fibrosis may cause abnormal vascularity such as reduced vascular density^59^ and leakier vasculature^60^, and subsequently, may impair therapeutic delivery and efficacy. Rather, insight into the pathways by which inflammation is resolved has highlighted novel opportunities to pharmacologically manipulate these processes as “resolution pharmacology”^57,61^. Impaired resolution leads to chronic inflammatory diseases such as rheumatoid arthritis, Crohn’s disease, and asthma^62^. Further studies are required to investigate whether impaired resolution is also involved in the pathophysiology of endometriosis, particularly in the fibrogenesis of endometriosis.

The effects of the highest dose of IL-1β (100 pg/mL) on cell proliferation, migration, collagen gel contraction, Col I, αSMA and MMP-I mRNA and/or protein expression of Ectopic-ES suggest that acute severe inflammation could markedly promote growth and tissue destruction and remodeling of already developed endometriotic tissues. Studies have suggested that the majority of endometriosis cases are not progressive^63^. Progression of the disease and appearance of specific symptoms rarely occurs in patients with asymptomatic rectovaginal endometriosis^64^. However, progression of the disease occurs in some symptomatic patients^65^ and to date, we do not know how to predict who will and will not progress^63^. Recent animal experiments showed that the stress-induced activation of hypothalamo-pituitary-adrenocortical (HPA) axis promoted the progression of endometriotic lesions^66,67^. IL-1β has been shown to induce strong sustained activation of the HPA axis^68–71^. Other pro-inflammatory cytokines including TNFα and IL-6 also activate the HPA axis, although they are much less potent than IL-1β^70,71^. There is considerable evidence for individual differences in the stress-induced HPA activation in humans^72^. Can acute severe inflammation hyperactivate the HPA axis in some patients, resulting in progression of the disease? Further epidemiological studies that investigate the link between acute severe inflammation and growth of endometriotic lesions should provide more information on the pathophysiology of endometriosis.

One of the limitations of the present study is that we do not know whether the present varying doses of IL-1β and/or TNFα (pg/mL range) reflect *in vivo* endometriotic tissues levels. Many previous *in vitro* experiments used much higher levels of IL-1β and/or TNFα (ng-µg/mL ranges) to elucidate the roles of these two proinflammatory cytokines in the pathophysiology of endometriosis^73–77^. However, previous studies reported much lower levels of IL-1β and/or TNFα in serum and peritoneal fluid of patients with endometriosis (pg/mL range)^78–82^. IL-1β production is extensively regulated in order to avoid highly detrimental effects of overproduction of IL-1β^83,84^. The margin between clinical benefit and undesirable pathogenic effects for IL-1 is exceedingly narrow^83,84^. IL-1β serum levels in the most severe IL-1β-mediated autoinflammatory diseases are only five-fold higher than in healthy controls^84^. Varying doses of IL-1β and/or TNFα (pg/mL range) in the present experiments are more likely to reflect *in vivo* endometriotic tissues levels. *In vivo* experiments are required to confirm the present preliminary *in vitro* findings. However, at the experimental level it is still challenging to mimic chronic inflammation in animal models as many inflammatory models are spurious and spontaneously resolve after several days or weeks of active inflammation^62^. Appropriate animal models to recapitulate human endometriosis and to investigate the effects of long-term cyclical inflammation by repeated bleeding of the endometrium and endometriosis on fibrosis may be *Old World nonhuman primates* with menstruation and spontaneous endometriosis, such as baboons and macaques^67,85,86^.

In conclusion, the present results suggest that low-grade inflammation promotes a fibrotic phenotype, whereas high-grade inflammation inactivates the fibrotic phenotype of endometriotic stromal cells. The present findings bring into question whether we should still continue to attempt anti-inflammatory treatment strategies in any patient with endometriosis. Anti-inflammatory treatment may prevent growth of endometriotic tissues in excessive inflammatory stages, whereas it may have deleterious effects on fibrotic endometriotic tissues in a low-grade inflammation setting.

## Materials and Methods

### Patients

Patients age 20–37 years undergoing laparoscopy for endometriosis were recruited at CHU Clermont-Ferrand for the present study. None of the women had received hormonal treatments, such as gonadotropin-releasing hormone agonists (GnRHa) or sex steroids, and none used intrauterine contraception for at least 6 months prior to surgery. Recruited patients had regular menstrual cycles (26–32 days) with confirmation of their menstrual history. Samples from 36 patients who had histological evidence of deep endometriosis and/or ovarian endometriosis, were used for the present analysis. Deep infiltrating endometriosis was defined as endometriosis located 5 mm under the peritoneal surface^87^. Patients with endometriotic ovarian cysts>3 cm in diameter were also included. In addition, menstrual-phase endometrial tissues were obtained from 16 patients with endometriosis and 8 patients who underwent tubal ligation or reversal as ‘true’ healthy controls.

Endometrial tissue biopsies were performed just prior to surgery using an endometrial suction catheter (Pipelle, Laboratoire CCD, Paris, France). Samples of endometrial and endometriotic tissue were immediately collected in Hanks’ balanced salt solution (Life Technologies, Cergy Pontoise, France). The clinical characteristics of patients are shown in Supplementary Table 1. The research protocol was approved by the Consultative Committee for Protection of Persons in Biomedical Research (CPP) of the Auvergne (France) region. All experiments were performed in accordance with approved guidelines and regulations. Informed written consent was obtained from each patient prior to tissue collection.

### Cell culture

Endometrial and endometriotic stromal cells were isolated as previously described^4–7,88^. Isolated cells were plated onto Primaria flasks (BD) in phenol red-free Dulbecco’s modified Eagle medium (DMEM)/F-12 containing 10% charcoal-stripped fetal bovine serum (FBS), 100 U/mL penicillin, 0.1 mg/mL streptomycin, and 0.25 µg/mL amphotericin B (Life Technologies, Cergy Pontoise, France) and incubated at 37 °C in 95% air/5% CO_2_. When the cells reached confluence, the first passages were used for experiments. Immunofluorescent staining (cytokeratin, vimentin, CD 10, αSMA)^4–7,88^ was performed to determine the purity of the isolated endometrial and endometriotic stromal cells as previously described^4–7,88^.

### Treatment of cells

For cell proliferation analyses, cells were incubated with IL-1β and/or TNFα at the indicated concentrations or vehicle only. For quantitative real-time reverse transcriptase (RT)-PCR and immunocytochemistry, to mimic inflammation followed by inflammatory resolution in tissue injury and repair, cells were incubated with pro-inflammatory cytokines (IL-1β and/or TNFα) at the indicated concentrations followed by an anti-inflammatory mediator (transforming growth factor-beta 1 [TGF-β1]: 5 ng/mL). For cell migration and collagen gel contraction assays, cells were incubated with IL-1β and/or TNFα at the indicated concentrations or vehicle only.

### RNA extraction, examination of RNA yield, and integrity and quantitative real-time RT-PCR

Endometrial or endometriotic stromal cells were seeded into 24-well plates (2.5 × 10^4^ cells per well) and were incubated with IL-1β and/or TNFα at the indicated concentrations or vehicle for 48 h. In another set, cells were pre-treated with IL-1β and/or TNFα at the indicated concentrations for 48 h, followed by TGF-β1 (5 ng/mL) stimulation for 48 h. Total RNA was extracted using the Qiagen RNeasy Mini Kit according to the manufacturer’s instructions (Qiagen, Courtaboef, France) as previously described^4,5,7^. RNA yield and integrity were analyzed using the RNA 6000 Pico kit and the Agilent Bioanalyzer 2100 (Agilent Technologies, Santa Clara, CA, USA) as previously described^4,5,7^. mRNA expression of Col I, MMP-1, and αSMA was measured by quantitative real-time RT-PCR with a Light Cycler (Roche, Mannheim, Germany) as previously described^4,5,7^. The procedure was repeated independently three times to ensure the reproducibility of the results^4,5,7^. All of the samples with a cycle threshold (Ct) coefficient of variation value>5% were retested^4,5,7^.

### Cell proliferation assays

Cell proliferation assays were performed using the CellTiter 96 AQueous One Solution Cell Proliferation Assay (MTS) (Promega, Charbonnières-les-Bains, France), as previously described^4,7,87^. Briefly, cells (5 × 10^3^ cells per well) were plated in triplicate in 96-well plates. After 48 h, cells were incubated with IL-1β and/or TNFα at the indicated concentrations or vehicle only for 48 h with 100 µL culture media (2% charcoal-stripped FBS) (Sigma-Aldrich). Then, 20 µL MTS solution were added to all wells and cells were incubated for 2 h at 37 °C. Absorbance was then read at 490 nm using a Multiskan microplate reader (Thermo Scientific, Illkirch, France). All values were normalized to the values obtained with vehicle-treated cells to control for unwanted sources of variation.

### *In vitro* migration assays

*In vitro* migration assays were performed using uncoated 24-well chambers/microfilters (BD), as previously described^4–7^,^89^. Briefly, after rehydration of the chambers, cells (2.5 × 10^4^ cells per chamber) in 500 µL phenol red-free DMEM/F12 without FBS (Life Technologies) were seeded onto the upper chamber. In the lower chamber, 750 µL phenol red-free DMEM/F12 plus 10% charcoal-stripped FBS (Life Technologies) were added. IL-1β and/or TNFα at the indicated concentrations or vehicle only was then added into the upper chamber. Cell motility/migration was measured as the number of cells that migrated from a defined area of the uncoated microfilter through micropores in 48 h. The micropore filters were stained with 0.5% crystal violet, and the number of cells that migrated through filters was counted in the entire area of each filter. To count cell numbers objectively, a computerized image analysis system consisting of a light microscope (Leica, Lyon, France) (X20 objective, X10 ocular) and a color charge-coupling device camera (Sony, Paris, France) were utilized. All experiments were performed in duplicate.

### Collagen gel contraction assay

Collagen gel contraction assays were performed as previously described^4,5^. Briefly, 24-well culture plates were coated with 1% BSA and incubated for 1 h at 37 °C to create a nonstick surface that prevented gels from attaching to the dishes. Endometriotic and endometrial stromal cells were seeded at a concentration of 2.5 × 10^5^ cells/mL into a 2.0-mg/mL Col I solution (BD, Le Pont de Claix, France) in PBS containing 0.023 N NaOH. The collagen/cell suspension was vortexed, and 500 µL per well was added to the BSA-coated plates. The solution was allowed to polymerize for 60 min at 37 °C. Five hundred microliters of culture media (2% charcoal-stripped FBS) containing either IL-1β and/or TNFα at the indicated concentrations or vehicle only were added to the three-dimensional solidified collagen gels, and plates were returned to the incubator. Collagen gel contraction was monitored over a period of 24 h, and the surface area of the contracted gels was measured at 24 h using ImageJ software (version 1.41) developed at the National Institute of Health. All experiments were performed in duplicate, because only limited number of Ectopic-ES were available for analysis.

### Immunofluorescence staining

Endometriotic or endometrial stromal cells were seeded onto glass coverslips (22 mm × 22 mm) (3 × 10^4^ cells per coverslip), and were incubated with 2 mL culture media (2% charcoal-stripped FBS) containing IL-1β and/or TNFα at the indicated concentrations or vehicle only for 48 h. In another set, cells were pre-treated with IL-1β, and/or TNFα at the indicated concentrations for 48 h, followed by TGF-β1 (5 ng/mL) stimulation for 48 h.

Double immunofluorescence staining for Col I (rabbit polyclonal, 1:500; Abcam, Cambridge, UK) and αSMA (1A4, 1:100; Merck Millipore) was performed in endometrial and endometriotic stromal cells, as previously reported^7,32^. Alexa Fluor 488 (green) goat anti-mouse IgG- and Alexa Fluor 594 (red) goat anti-rabbit IgG-conjugated antibodies (Life Technologies) were used as secondary antibodies. Cell nuclei were stained with 4, 6-diamidino-2-phenylindole (DAPI) (Life Technologies). Slides were analyzed with a Leica TCS SPE confocal laser-scanning microscope (Leica Microsystems, Nanterre, France). The percentage of cells with αSMA + stress fibers, and the percentage of Col I + cells among the total number of DAPI-stained nuclei were calculated from 10 random high-power (x400) fields through each section, as previously reported^7,32^.

### Statistical analysis

The STATA program version 12 (StataCorp, College Station, TX, USA) was used for statistical analysis. Comparisons between groups were made using one-way analysis of variance (ANOVA) following Scheffé’s method, the Mann-Whitney *U* test or the Wilcoxon matched pairs signed-ranks test. According to the results of the present cell proliferation assays, effects of the highest dose of IL-1β (100 pg/mL) on cell migration, collagen gel contraction, mRNA and/or protein expression of Col I, MMP-1, and αSMA in menstrual endometrial stromal cells of patients with endometriosis (M-ES-endo) and those of healthy fertile women (M-ES-healthy) were excluded for further analyses, due to markedly reduced cell viability. Statistical significance was defined as p < 0.05.

## Supplementary information

Supplementary information.

Supplementary information2.

Supplementary information3.
