# Supplementary material for: Dose-dependent pro- or anti-fibrotic responses of endometriotic stromal cells to interleukin-1β and tumor necrosis factor α
Source: Sci Rep. 2020 Jun 11;10:9467. doi: 10.1038/s41598-020-66298-x (PMC7289797; doi:10.1038/s41598-020-66298-x)

Supplementary Figure S1

IL-1β

A: 4h

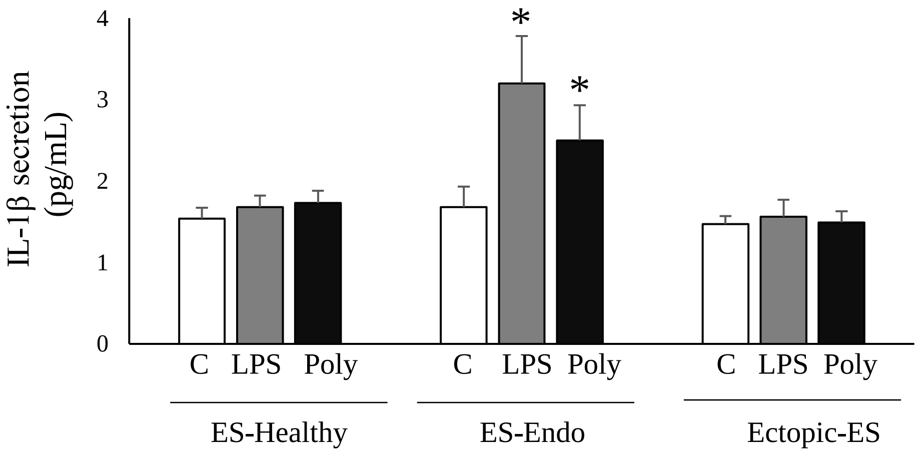

B: 24h

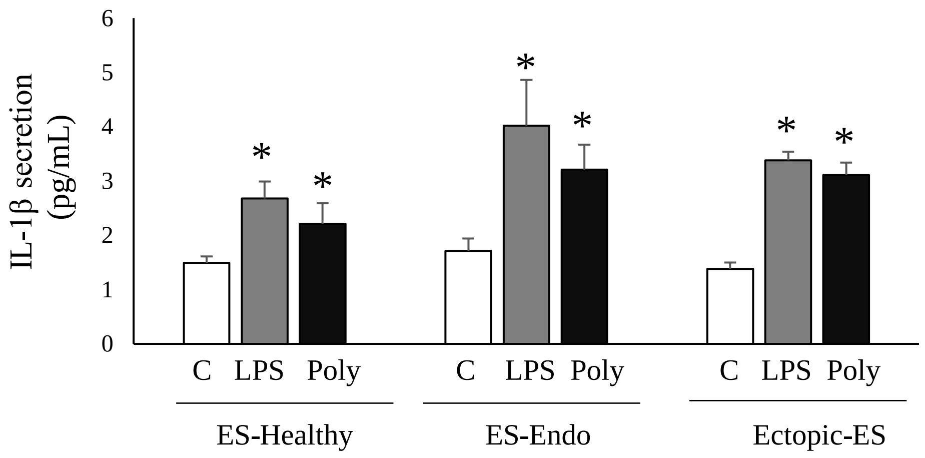

TNFα

C: 4h

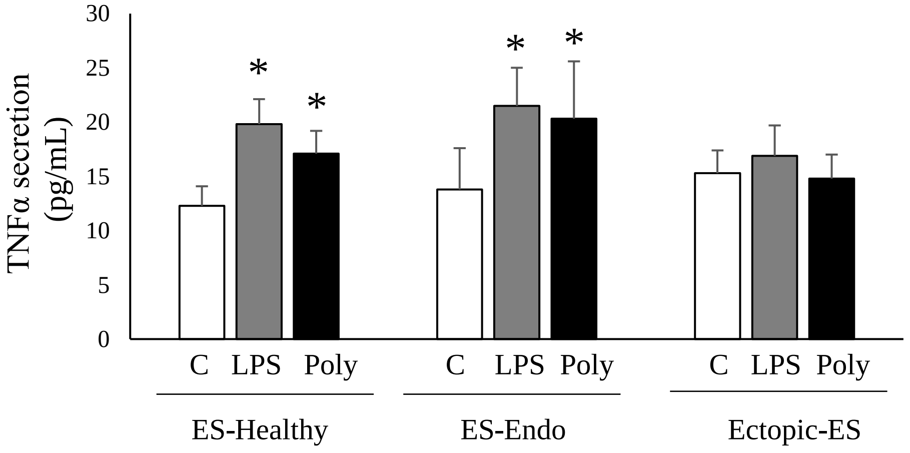

D: 24h

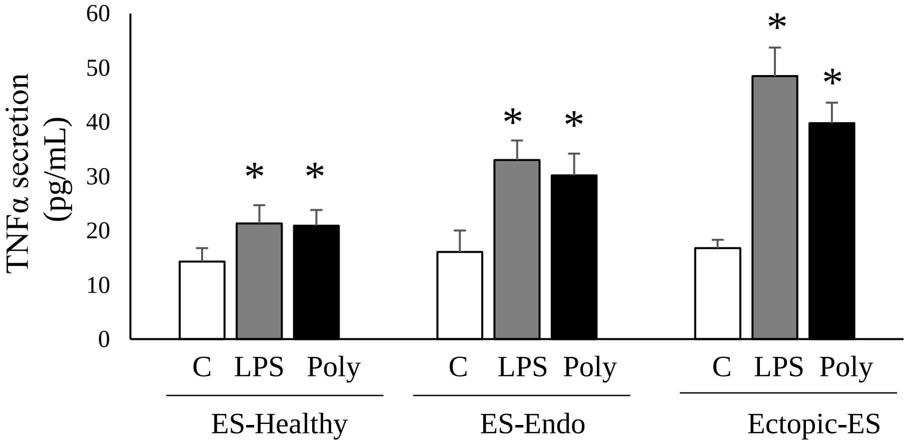

Supplementary Figure S2

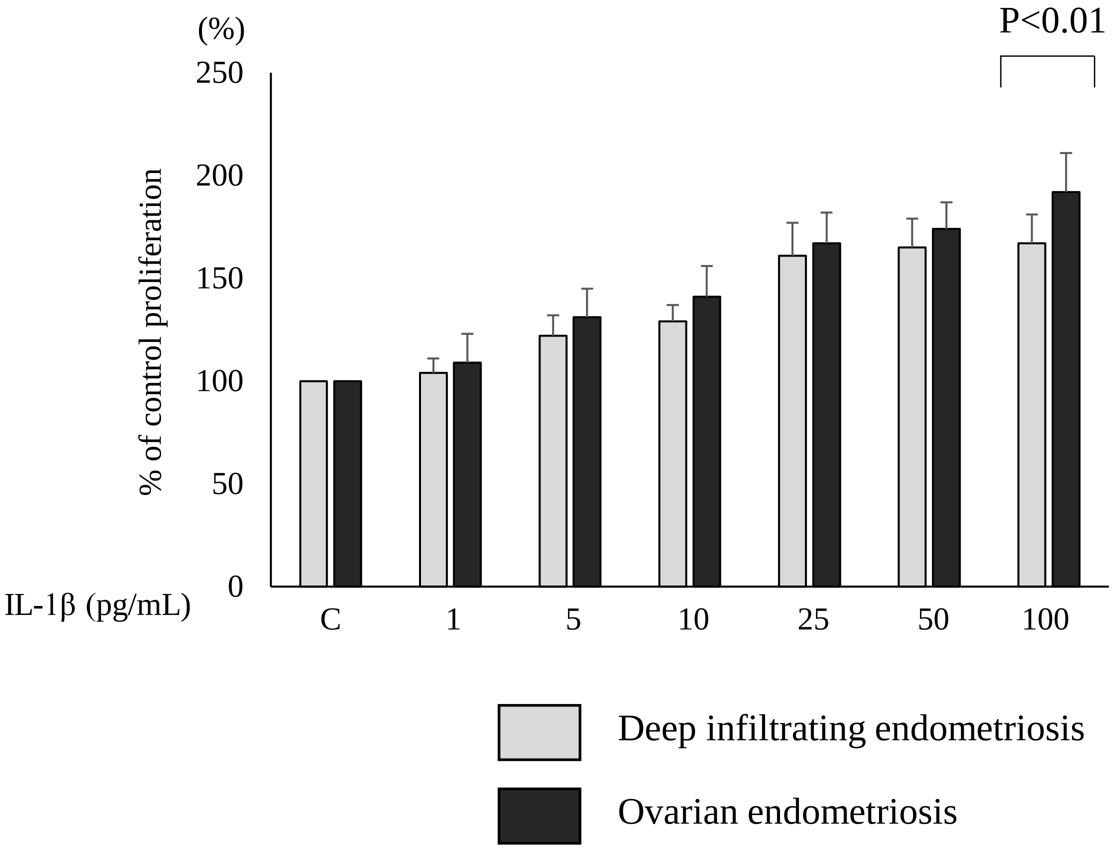

Supplementary Figure S3

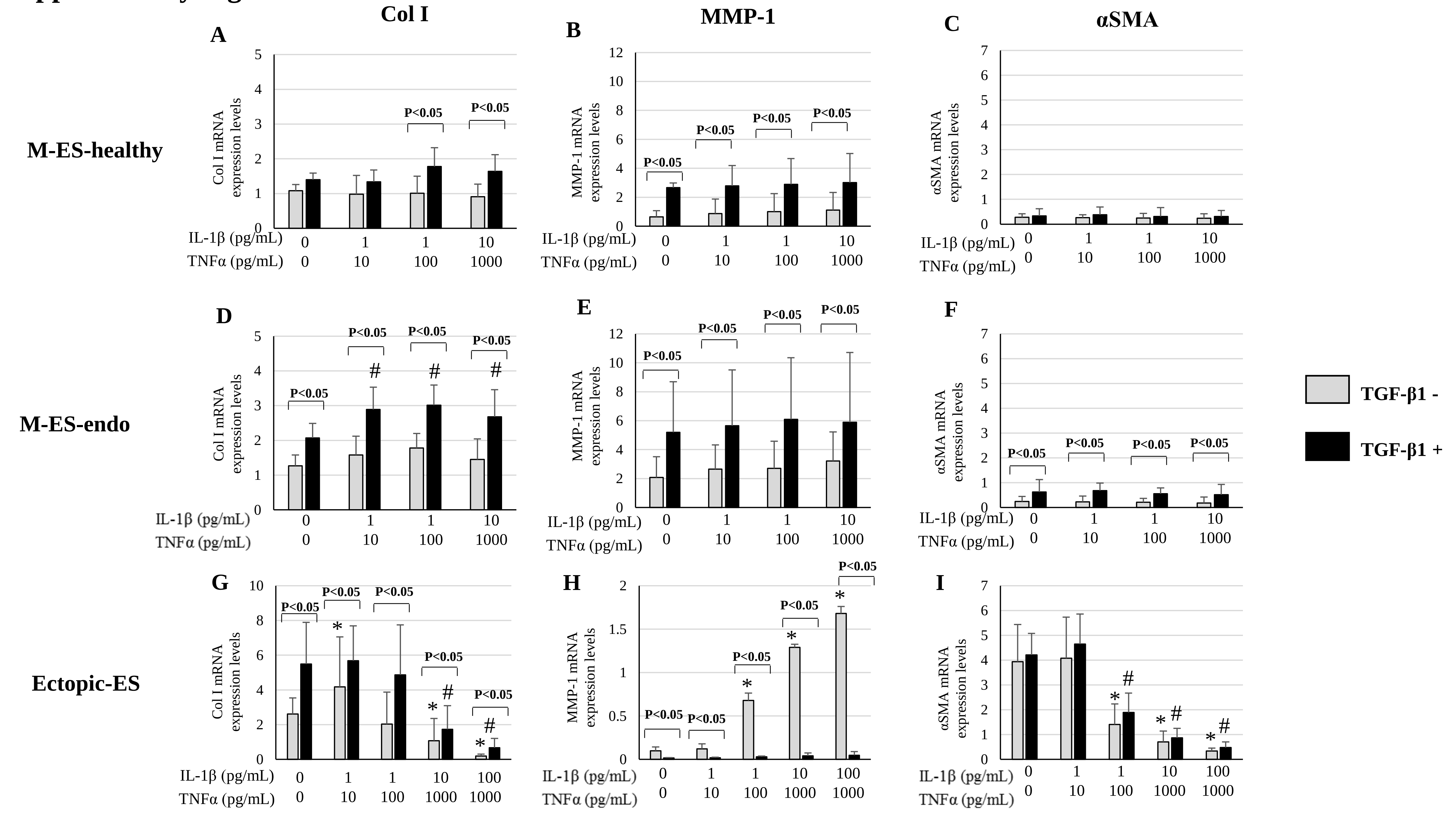

Supplementary Figure S4

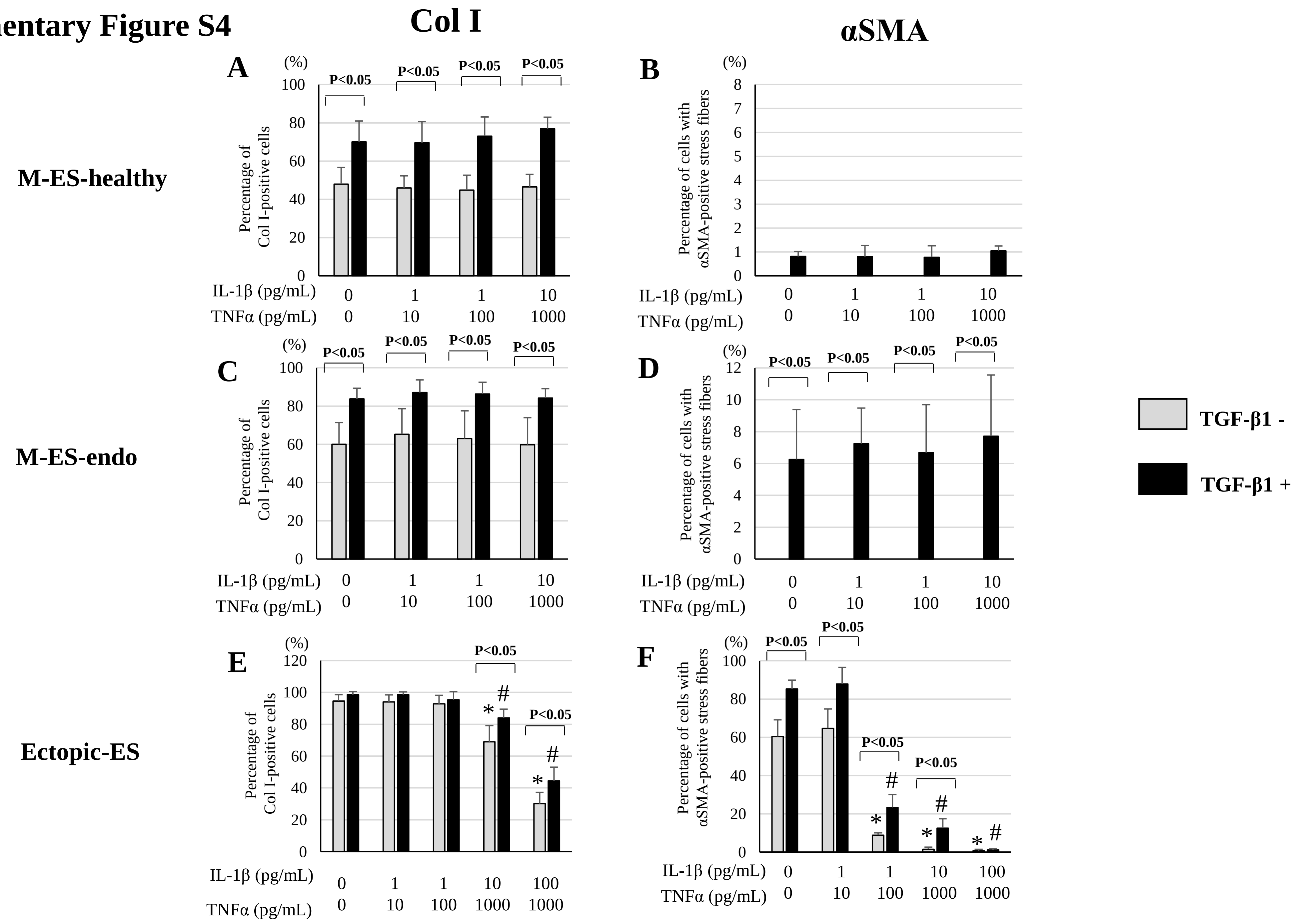

Supplementary Figure S5

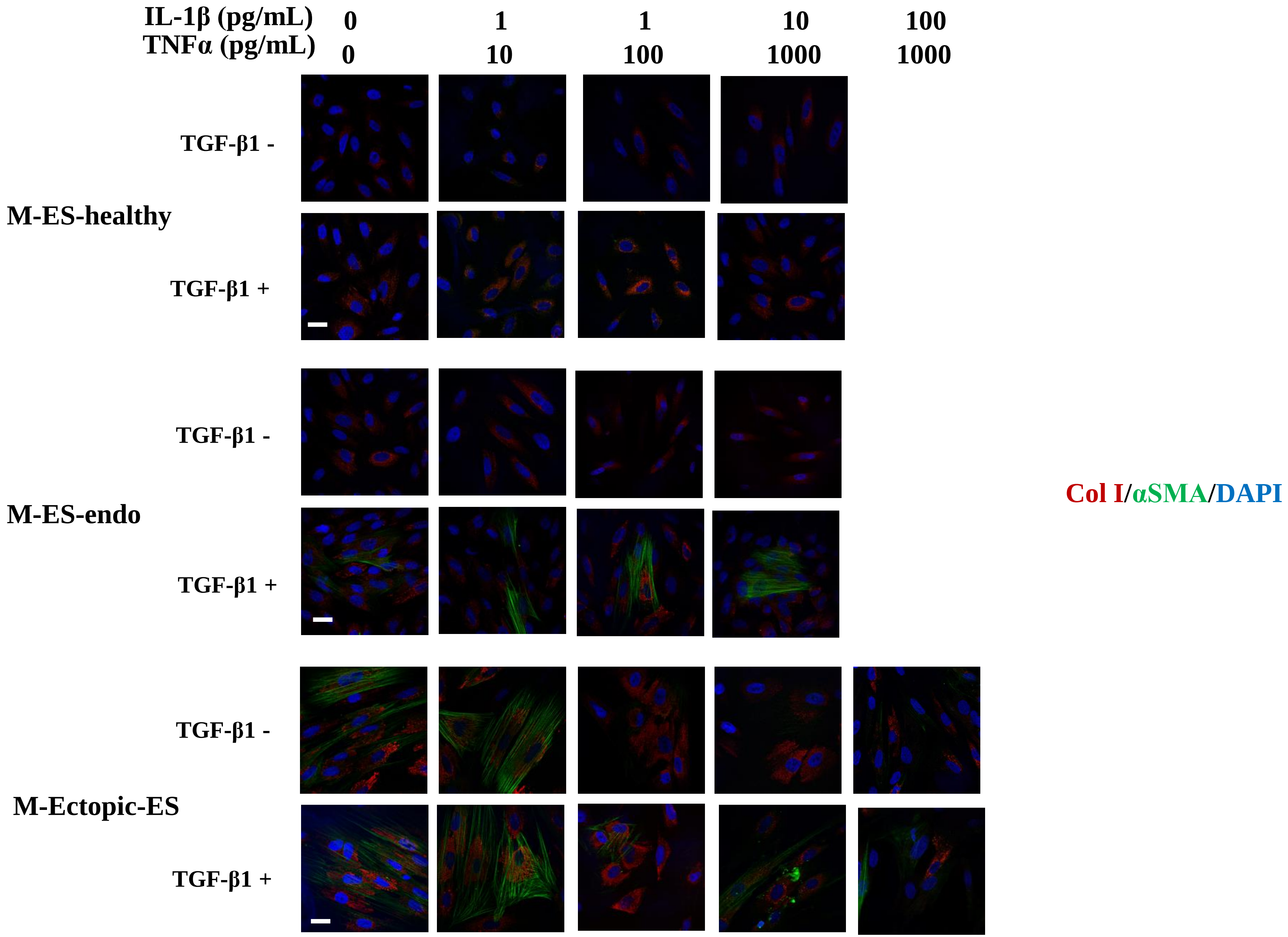

Supplement: Supplementary file 1 — Supplementary information. [file 41598_2020_66298_MOESM1_ESM.pdf]
