# Supplementary material for: Dose-dependent pro- or anti-fibrotic responses of endometriotic stromal cells to interleukin-1β and tumor necrosis factor α
Source: Sci Rep. 2020 Jun 11;10:9467. doi: 10.1038/s41598-020-66298-x (PMC7289797; doi:10.1038/s41598-020-66298-x)
Supplement: Supplementary file 2 — Supplementary information2. [file 41598_2020_66298_MOESM2_ESM.pdf]

Dose-dependent pro- or anti-fibrotic responses of endometriotic stromal cells to interleukin-1β and tumor necrosis factor α

Running title: Inflammation and endometriosis

Sachiko Matsuzaki [1, 2, 3]

Jean-Luc Pouly [1, 2]

Michel Canis [1, 2]

[1] CHU Clermont-Ferrand, Chirurgie Gynécologique, Clermont-Ferrand, France

[2] Université Clermont Auvergne, Institut Pascal, UMR6602, CNRS/UCA/SIGMA, Clermont-Ferrand, France

[3] Corresponding Author: Sachiko Matsuzaki

CHU Clermont-Ferrand, Chirurgie Gynécologique, 1, Place Lucie et Raymond Aubrac, 63003 Clermont-Ferrand, France, Tel: +33 4 73 75 01 38, E-mail: sachikoma@aol.com

Supplementary Note

Varying doses of IL-1β and/or TNFα in the present study

Many previous in vitro experiments used very high levels of IL-1β and/or TNFα (ng-μg/mL ranges) to elucidate the roles of these two proinflammatory cytokines in the pathophysiology of endometriosis.[1-5] However, it is not clear whether such high levels of IL-1β and/or TNFα could be involved in the pathophysiology of endometriosis. Previous studies reported much lower levels of IL-1β and/or TNFα in serum and peritoneal fluid of patients with endometriosis (pg/mL range).[6-10] Although proinflammatory cytokines are produced predominantly by activated macrophages, a previous in vitro study showed that peritoneal macrophages from patients with endometriosis produced TNFα in the pg/ml range following stimulation with LPS. High levels of IL-1β and/or TNFα (ng-μg/mL ranges) in previous experiments [1-5] may not be recapitulated in the in vivo environment of patients with endometriosis.

Thus, first, we measured active IL-1β and TNFα secretion of endometriotic stromal cells, and menstrual endometrial stromal cells of patients with endometriosis and those of healthy control women, after stimulation with LPS or Poly I:C, to estimate potential pathophysiological ranges of IL-1β and TNFα in endometriotic tissues. In endometriosis, as in many other fibrotic disorders,[11] the inflammatory triggers remain unknown. Thus, in the present in vitro experiments, inflammation was non-specifically triggered by stimulation of innate immunity with either lipopolysaccharide (LPS) or polyinosinic:polycytidylic acid (Poly I:C).[11] Both toll-like receptor (TLR)-3 and -4 recognize dying/dead cells.[12] During menstruation, dying endometrial cells may activate TLRs, including TLR-3 and -4, in endometrial cells. Furthermore, studies have shown that sustained TLR signaling contributes to the development of many chronic inflammatory and autoimmune diseases.[13, 14] Thus, in the present study, inflammation was triggered by acute (4-h) and chronic (24-h) stimulation with either LPS or Poly I:C.

The present ELISA analyses suggested that TLR activation may induce low-grade local inflammation in endometriotic and menstrual endometrial stromal cells. According to the present ELISA analysis results as well as those of previous in vivo studies,[6-10] we further evaluated the effects of varying ranges of IL-1β and TNFα (pg/mL range) on fibrosis in endometriosis.

References

1. Lebovic, D.I. et al. Induction of an angiogenic phenotype in endometriotic stromal cell cultures by interleukin-1beta. *Mol. Hum. Reprod.* **6**, 269-275 (2000).
2. Tamura, M. et al. Interleukin-1beta elevates cyclooxygenase-2 protein level and enzyme activity via increasing its mRNA stability in human endometrial stromal cells: an effect mediated by extracellularly regulated kinases 1 and 2. *J. Clin. Endocrinol. Meta.* **87**, 3263-3273 (2002).
3. Wu, M.H. et al. Distinct regulation of cyclooxygenase-2 by interleukin-1beta in normal and endometriotic stromal cells. *J. Clin. Endocrinol. Metab.* **90**, 286-295 (2005).
4. Cao, W.G., Morin, M., Metz, C., Maheux, R. & Akoum, A. Stimulation of macrophage migration inhibitory factor expression in endometrial stromal cells by interleukin 1, beta involving the nuclear transcription factor NFkappaB. *Biol. Reprod.* **73**, 565-570 (2005).
5. McKinnon, B.D., Evers, J., Bersinger, N.A. & Mueller, M.D. Induction of the neurokinin 1 receptor by TNFα in endometriotic tissue provides the potential for neurogenic control over endometriotic lesion growth. *J. Clin. Endocrinol. Metab.* **98**, 2469-2477 (2013).
6. Cheong, Y.C. et al. IL-1, IL-6 and TNF-alpha concentrations in the peritoneal fluid of women with pelvic adhesions. Hum Reprod. **17**, 69-75 (2002).
7. Richter, O.N., Dorn, C., Rösing, B., Flaskamp, C. & Ulrich, U. Tumor necrosis factor alpha secretion by peritoneal macrophages in patients with endometriosis. *Arch. Gynecol. Obstet.* **271**, 143-147 (2005).
8. Kalu, E. et al. Cytokine profiles in serum and peritoneal fluid from infertile women with and without endometriosis. *J. Obstet. Gynaecol. Res.* **33**, 490-495 (2007).

9. McKinnon, B., Bersinger, N.A., Wotzkow, C. & Mueller, M.D. Endometriosis-associated nerve fibers, peritoneal fluid cytokine concentrations, and pain in endometriotic lesions from different locations. *Fertil. Steril.* **97**, 373-380 (2012).
10. Nematian, S.E. et al. Systemic Inflammation Induced by microRNAs: Endometriosis-Derived Alterations in Circulating microRNA 125b-5p and Let-7b-5p Regulate Macrophage Cytokine Production. *J. Clin. Endocrinol. Metab.* **103**, 64-74 (2018).
11. Wynn, T.A. & Ramalingam, T.R. Mechanisms of fibrosis: therapeutic translation for fibrotic disease. *Nat. Med.* **18**, 1028–1040 (2012).
12. Rock, K.L., Lai, J.J. & Kono, H. Innate and adaptive immune responses to cell death. *Immunol. Rev.* **243**, 191-205 (2011).
13. Anwar, M.A., Basith, S. & Choi, S. Negative regulatory approaches to the attenuation of Toll-like receptor signaling. *Exp. Mol. Med.* **45**, e11 (2013).
14. Gao, W., Xiong, Y., Li, Q. & Yang, H. Inhibition of Toll-Like Receptor Signaling as a Promising Therapy for Inflammatory Diseases: A Journey from Molecular to Nano Therapeutics. *Front. Physiol.* **8**, 508; 10.3389/fphys.2017.00508 (2017).

Supplementary Methods

Treatment of cells

For enzyme-linked immunosorbent assay (ELISA) analyses, cells were incubated with either LPS (100 ng/mL), Poly I:C (10 µg/mL), or vehicle alone for 4 h or 24 h. Human recombinant IL-1β protein (R&D Systems, Lille, France) and TNFα protein (R&D Systems) were dissolved in phosphate-buffered saline (PBS) containing 0.1% bovine serum albumin (BSA). LPS (Sigma-Aldrich), Poly I:C (Sigma-Aldrich), or adenosine triphosphate (ATP; Sigma-Aldrich) were dissolved in phenol red-free DMEM:Nutrient Mixture F-12 (DMEM/F-12) (Life Technologies).

Cytokine quantification

Secreted IL-1β and TNFα levels in culture supernatants were assayed in duplicate and quantified by ELISA with DuoSet ELISA kits (R&D Systems) according to the manufacturer's instructions. The lower detection limits were 1.0 pg/mL (IL-1β) and 6.23 pg/mL (TNFα). Briefly, cells (3×10^4 cells per well) were plated in 24-well plates. After 48 hours, LPS, Poly I:C, or vehicle were added with 500 µL culture media (2% charcoal-stripped FBS) (Sigma-Aldrich) and incubated for 4 h or 24 h, and supernatants were collected. We utilized a well-established LPS or Poly I:C (1st signal; priming)/ATP (a damage-associated molecular pattern [DAMP]) (2nd signal; NLR activation) stimulation protocol to measure active IL-1β secretion after activation of the NLRP3 inflammasome.[1] Cells were incubated with 3 mM ATP for the last 15 min based on the method of Stoffels et al.[1] to measure active IL-1β secretion. Absorbance was read at 450/540 nm using a Multiskan microplate reader (Thermo Scientific). The concentration (pg/mL) was normalized to total protein content (pg/µg total protein) to control for unwanted sources of variation. Intra-assay precision was 4.3% (IL-1β) and 5.4% (TNFα), and inter-assay precision was 7.4% (IL-1β) and 8.9% (TNFα).

References

1. Stoffels, M. et al. ATP-Induced IL 1β Specific Secretion: True Under Stringent Conditions. *Front. Immunol.* **6**, 54; 10.3389/fimmu.2015.00054 (2015).

Supplementary Results

Effects of LPS or Poly I:C stimulation on IL-1β and TNFα secretion in endometrial and endometriotic stromal cells

Neither LPS nor Poly I:C stimulation had a significant effect on IL-1β secretion at 4 h in M-ES-healthy (Supplementary Figure S1A). In contrast, after 24-h chronic stimulation with either LPS or Poly I:C, IL-1β secretion significantly increased in M-ES-healthy (Supplementary Figure S1B). Both LPS and Poly I:C stimulation significantly increased IL-1β secretion in M-ES-endo at 4 h (Supplementary Figure S1A) and 24 h (Supplementary Figure S1B). In both M-ES-healthy and M-ES-endo, both LPS and Poly I:C stimulation significantly increased TNFα secretion at 4 h (Supplementary Figure S1C) and 24 h (Supplementary Figure S1D). In ectopic endometriotic cells (Ectopic-ES), neither LPS nor Poly I:C stimulation had a significant effect on IL-1β (Supplementary Figure S1A) or TNFα (Supplementary Figure S1C) secretion at 4 h. In contrast, after 24-h chronic stimulation with either LPS or Poly I:C, IL-1β (Supplementary Figure S1B) and TNFα (Supplementary Figure S1D) secretion significantly increased.

Supplementary Figure Legends

Supplementary Figure S1: Effects of LPS (100 ng/mL) or Poly I:C (10 μg/mL) stimulation for 4 h or 24 h on interleukin-1β (IL-1β) (A, B) secretion or tumor necrosis factor α (TNFα) (C, D) secretion in menstrual endometrial stromal cells of healthy fertile women (M-ES-healthy) and those of patients with endometriosis (M-ES-endo), and endometriotic stromal cells (Ectopic-ES)

Numerical values are presented as the mean + SD.
*: $p<0.05$ versus control (vehicle alone) within the same group.

M-ES-healthy: n=6
M-ES-endo: n=8
Ectopic-ES: n=10

Supplementary Figure S2

Effects of IL-1β on cell proliferation of Ectopic-ES derived from deep infiltrating endometriosis versus ovarian endometriosis.

Cells were incubated for 48 h at the indicated concentrations.
Percent cell proliferation was calculated as percent of vehicle control after 48-h treatment.
Numerical values are presented as the mean ± SD.

C: control (vehicle alone)

Deep infiltrating endometriosis: n=8
Ovarian endometriosis: n=8

Supplementary Figure S3: Effects of combinations of IL-1β and TNFα with or without TGF-β1 on mRNA expression of Col I (A, D, G), MMP-1 (B, E, H), and αSMA (C, F, I) in M-ES-healthy (A-C), M-ES-endo (D-F), and Ectopic-ES (G-I).

Cells were incubated at the indicated concentrations.

Expression levels of Col-I, MMP-1, and αSMA mRNAs are given relative to the expression level of the reference gene, glyceraldehyde 3-phosphate dehydrogenase (GAPDH).

Numerical values are presented as the mean + SD.

*: $p<0.05$ versus control (vehicle alone).

#: $p<0.05$ versus TGF-β1 alone

Effects of the highest dose of IL-1β (100 pg/mL) on mRNA expression of Col I, MMP-1, and αSMA in M-ES-healthy and M-ES-endo were excluded for further analyses, due to markedly reduced cell viability.

Because there were no significant differences in either Col I or αSMA protein expression between vehicle-treated control after 48h and 96h, results of vehicle-treated control after 96h were not shown.

M-ES-healthy: n=8

M-ES-endo: n=16

Ectopic-ES: n=22

Supplementary Figure S4: Effects of combinations of IL-1β and TNFα with or without TGF-β1 on Col I protein expression (A, C, E) and αSMA+ stress fibers (B, D, F) in M-ES-healthy (A, B), M-ES-endo (C, D), and Ectopic-ES (E, F).

(A-F) The percentage of cells with Col I+ or αSMA+ stress fibers after stimulation with IL-1β or TNFα alone or following TGF-β1 5 ng/mL stimulation in M-ES-healthy, M-ES-endo, and Ectopic-ES.

*: $p<0.05$: versus control (vehicle alone)

#: $p<0.05$ versus TGF-β1 alone

Numerical values are presented as the mean ± SD.

M-ES-healthy: n=8

M-ES-endo: n=16

Ectopic-ES: n=22

Effects of the highest dose of IL-1β (100 pg/mL) on protein expression of Col I and αSMA in M-ES-healthy and M-ES-endo were excluded for further analyses, due to markedly reduced cell viability.

Because there were no significant differences in either Col I or αSMA protein expression between vehicle-treated control after 48h and 96h, results of vehicle-treated control after 96h were not shown.

Supplementary Figure S5: Representative photomicrographs of double immunofluorescence staining for Col I and αSMA in M-ES-healthy, M-ES-endo, and Ectopic-ES after stimulation with IL-1β and/or TNFα alone or following TGF-β1 5 ng/mL stimulation. Scale bar: 50 μm.

M-ES-healthy: n=8

M-ES-endo: n=16

Ectopic-ES: n=22

Effects of the highest dose of IL-1β (100 pg/mL) on protein expression of Col I and αSMA in M-ES-healthy and M-ES-endo were excluded for further analyses, due to markedly reduced cell viability.
