# Supplementary material for: Dose-dependent pro- or anti-fibrotic responses of endometriotic stromal cells to interleukin-1β and tumor necrosis factor α
Source: Sci Rep. 2020 Jun 11;10:9467. doi: 10.1038/s41598-020-66298-x (PMC7289797; doi:10.1038/s41598-020-66298-x)
Supplement: Supplementary file 3 — Supplementary information3. [file 41598_2020_66298_MOESM3_ESM.pdf]

Supplementary Table S1. Clinical characteristics of patients

	Endometriosis	Healthy Fertile women
No of cases	42[c]	8
Age [a]	32.0 (21-37)	36.5 (30-38)
Parity [a]	0 (0-1)	3 (3-7)
rASRM stage[b]		
I	15	
II	10	
III	9	
IV	8	

[a] Median (range)

[b] Revised American Society for Reproductive Medicine classification (rASRM) (American Society for Reproductive Medicine, 1997).

[c] Among 42 patients, paired endometriotic and menstrual endometrial tissues from 10 patients were collected
